# Intracranial Hemorrhage After Reperfusion Therapies in Acute Ischemic Stroke Patients

**DOI:** 10.3389/fneur.2020.599908

**Published:** 2020-12-11

**Authors:** Benjamin Maïer, Jean Philippe Desilles, Mikael Mazighi

**Affiliations:** ^1^Interventional Neuroradiology Department, Hôpital Fondation Adolphe de Rothschild, Paris, France; ^2^Université de Paris, Paris, France; ^3^Laboratory of Vascular Translational Science, INSERM U1148, Paris, France

**Keywords:** acute ischemic stroke, intracranial hemorrhage, thrombolysis, blood pressure, endovascular treatment, thrombectomy, disabilility, mortality

## Abstract

Reperfusion therapies are the mainstay of acute ischemic stroke (AIS) treatments and overall improve functional outcome. Among the established complications of intravenous (IV) tissue-type plasminogen activator (tPA), intracranial hemorrhage (ICH) is by far the most feared and has been extensively described by seminal works over the last two decades. Indeed, IV tPA is associated with increased odds of any ICH and symptomatic ICH responsible for increased mortality rate during the first week after an AIS. Despite these results, IV tPA has been found beneficial in several pioneering randomized trials and improves functional outcome at 3 months. Endovascular therapy (EVT) combined with IV tPA for AIS patients consecutive to an anterior circulation large-vessel occlusion does not increase ICH occurrence. Of note, EVT following IV tPA leads to significantly higher rates of early reperfusion than with IV tPA alone, with no difference in ICH, which challenges the paradigm of reperfusion as a major prognostic factor for ICH complications. However, several blood biomarkers (glycemia, platelet and neutrophil count), clinical factors (age, AIS severity, blood pressure management, diabetes mellitus), and neuroradiological factors (cerebral microbleeds, infarct size) have been identified as risk factors for ICH after reperfusion therapy. In the years to come, the ultimate goal will be to further improve either reperfusion rates and functional outcome, while reducing hemorrhagic complications. To this end, various approaches being investigated are discussed in this review, such as blood-pressure control after reperfusion or the use of new antiplatelet agents as an adjunct to IV tPA and exhibit reduced hemorrhagic potential during the early phase of AIS.

## Introduction

To date, reperfusion therapies represent the mainstay of acute ischemic stroke (AIS) treatments (1, 2). Reperfusion can be performed pharmacologically by the use of intravenous recombinant human tissue-type plasminogen activator (IV tPA; alteplase, Boehringer Ingelheim, Germany) within the first 4.5 h after stroke onset (3), and since 2015, by endovascular therapy (EVT) in case of an anterior circulation large-vessel occlusion (LVO) (4). These different treatments have been found effective in reducing 3 month neurological disability. Indeed, in a meta-analysis of individual data from randomized controlled trials (RCTs), at 3 months, 32.9% of patients with IV tPA within 3 h after AIS onset had a modified Rankin Scale score 0–1 vs. 23.1% of the placebo group (5). Similarly, EVT following IV tPA vs. IV tPA alone in a setting of LVO of the anterior circulation significantly reduced disability at 3 months (4).

However, these treatments are by no means entirely free of complications, with intracranial hemorrhage (ICH) the most feared. Hemorrhagic complications after reperfusion therapies include a broad spectrum of severity between small petechial hemorrhagic infarcts (HIs) to parenchymal hematomas (PHs) (6–8). ICH, especially with PH, is associated with increased morbidity and mortality (9, 10). This explains in part why ICH and especially symptomatic ICH (sICH) are mandatory safety outcomes of most AIS RCTs. Nevertheless, the ICH description remains challenging, given its several different classifications and timeframe assessment (8). sICH and asymptomatic ICH after AIS have been differentiated, but the prognostic significance of asymptomatic ICH remains largely unknown. In this review, we discuss the implications of ICH after IV tPA and EVT. After examining the different clinical, radiological, and biological baseline characteristics associated with increased ICH occurrence, we review possible modifiable factors and future therapeutic approaches.

## ICH After Intravenous TPA Therapy

### Background

The serine protease tPA is mainly synthesized in endothelial cells and scarcely present in blood in the physiological condition. It converts the proenzyme plasminogen into plasmin. The active form of plasmin cleaves fibrin strands into small fibrin degradation products leading to fibrinolysis. This fibrinolysis cascade is tightly regulated under physiological conditions. Especially, at a physiological concentration, tPA can only activate plasminogen into plasmin when associated with fibrin, thus forming the ternary fibrinolysis complex responsible for a highly local fibrinolysis activation (11). However, numerous studies of IV tPA therapy for acute myocardial infarction have found that with tPA in the therapeutic concentration range, plasminogen can also be converted to plasmin in contact with circulating fibrinogen owing to the incomplete fibrin specificity of tPA. Therefore, plasmin can degrade fibrinogen, thus leading to a corresponding fibrinogen consumption and the formation of fibrinogen products (12, 13). The formation of fibrinogen degradation products with anticoagulant properties, called fibrinogen degradation coagulopathy, was linked to a surplus of hemorrhagic complications in acute myocardial infarction studies (14). In a recent study of AIS patients receiving IV tPA, 20% of patients showed fibrinogen degradation coagulopathy defined as a fibrinogen level decrease ≥200 mg/dL at 6 h after IV tPA infusion, which was associated with increased rate of hemorrhagic complications (15).

The natural history of AIS by itself is associated with the occurrence of delayed ICH, usually called hemorrhagic transformation or hemorrhagic infarction. In the European Cooperative Acute Stroke Study (ECASS) 3 trial, the frequency of any ICH in the placebo group reached 17.6%, with 3.5% being sICH according to the National Institute of Neurological Disorders and Stroke (NINDS) definition (3). Notably, the ICH risk in the setting of AIS without IV tPA therapy remains low, with mainly small petechial HIs associated with baseline stroke severity (16) but with limited, if any, impact on 3 month functional outcome in the ECASS 1 and 2 trials (17–19). These HIs were statistically independent of tPA use in the ECASS 1 and 2 trials and had a paradoxical lower proportion in the tPA group as compared with the placebo group (8). Thus, this ICH could be mainly due to the natural history of AIS and not the IV tPA use (19–22). In the late 1980s and early 1990s, when IV tPA was not approved for AIS and primary management was restricted to clinical monitoring, these HIs were frequently described (43%) and usually associated with severe AIS criteria such as brain edema, mass effect, blood–brain barrier disruption, and baseline neurological severity (22). In the setting of LVO AIS, the proportion of these HIs could even reach 50%, and they were associated with early hypodensities on baseline computed tomography (CT) scans (20). The pathophysiological mechanisms underlying HI development are still not elucidated. Reperfusion has long been cited as a main causal factor for the occurrence of HIs or ICH in general, along with blood pressure or hyperglycemia (23). However, this paradigm is now clearly questioned because of the lack of increased ICH risk observed after EVT-induced reperfusion, as discussed below.

### ICH and sICH Definitions

As the most severe complication of IV tPA, ICH defined in the setting of AIS is crucial because it involves key safety outcomes in RCTs and may help neurologists in the everyday management of AIS. ICH can be defined clinically (symptomatic vs. asymptomatic), radiologically (HIs vs. PHs) but also within the timeframe of assessment (early vs. late) (8). The notion of sICH in the setting of AIS was first introduced by Levy et al., requiring contemporaneous neurological worsening or a new mass effect on brain CT scan (24, 25). In the NINDS trial (parts 1 and 2), the clinical aspect of ICH was taken into account by the notion of a “temporally related neurologic deterioration,” without further details (6, 8). The Prolyse in Acute Cerebral Thromboembolism (PROACT) II trial, assessing the efficacy of intra-arterial prourokinase for AIS, was the first trial to specifically define the meaning of clinical deterioration after treatment, as an increase from baseline of four points in the National Institutes of Health Stroke Scale (NIHSS) score (8, 26). Because several conditions may increase the NIHSS score in the setting of AIS (e.g., epilepsy, mass effect, ischemic lesion growth), further trials specified ICH as the presumed responsible cause of neurological deterioration (≥4 points in NIHSS score). The rationale for distinguishing sICH and asymptomatic ICH is prognosis. First, and as mentioned above, asymptomatic ICH usually involves HI lesions as opposed to PH lesions, without any impact on 3 month functional outcome (19). However, this last point must be considered with caution in light of recent studies highlighting the association between asymptomatic ICH after EVT and worse clinical outcome (27, 28). Second, such distinction provides a practical and useful tool for RCTs to assess the safety related to IV tPA use (8).

The radiological distinction was introduced in the early 1990s and first distinguished HI and PH on follow-up CT scan (7). These lesions were used in the NINDS study as “acute infarction with punctate or variable hypodensity/hyperdensity, with an indistinct border within the vascular territory” for HIs and “typical homogeneous, hyperdense lesion with a sharp border with or without edema or mass effect” for PHs (29). In the ECASS trials, these definitions were implemented and improved with the distinction between HI1 and HI2 and PH1 and PH2 (Table 1). Therefore, the ECASS definition includes the ICH mass effect that was not considered in the NINDS definition (<30% or ≥30% of the infarct area with or without space-occupying effect) depending on the PH type. PH1 is associated with risk of early neurological deterioration but not disability or death (19). In contrast, PH2 is strongly associated with early neurological deterioration but also with 3 month disability and death (19). In the ECASS 1 trial, PH1 was not associated with neurological deterioration, disability, or death, but PH2 was strongly associated with early neurological deterioration [odds ratio (OR) = 32.3, 95% confidence interval (CI) = 13.4–77.7] and 3 month death (OR = 18, 95% CI = 8.05–40.1) (30). As discussed below, PH2 is strongly associated with tPA use and increases with tPA dose (3-fold for 0.9 mg/kg and 3.6-fold for 1.1 mg/kg) (8). Given its tremendous clinical significance, the Safe Implementation of Thrombolysis in Stroke-Monitoring Study (SITS-MOST) definition used local or remote PH2 on the 22 to 36 h posttreatment CT scan as a surrogate marker for sICH (Table 1) (31). More recently, the ECASS 4, the Extending the Time for Thrombolysis in Emergency Neurological Deficits (EXTEND), and the Echoplanar Imaging Thrombolytic Evaluation trials also used PH2 for defining sICH, when associated with an increase of four points in NIHSS score (32). Finally, the ICH and sICH classifications may differ regarding the timeframe of evaluation. In brief, the NINDS, PROACT II, and ECASS 1 trials assessed early ICH within 36 h after stroke onset (6, 8, 17, 26). In contrast, other trials considered a delayed timeframe for ICH assessment: within 7 days (3, 33). As discussed, delayed assessment could result in poorer correlations with tPA use, notably owing to its known short half-life and also in the context of EVT (8). In this context, the Heidelberg bleeding classification was developed during the XII Thrombolysis Symposium on Thrombolysis, Thrombectomy and Ischemic Stroke treatment with the aim to propose a revised classification that specifies, in a single format, ICH topography (intraparenchymal, subdural, and subarachnoid, to take into account EVT devices complications); localization (within the infarct or remote); symptomatic or asymptomatic aspects; and type of ICH (HI1, 2; PH1, 2) to harmonize everyday practice and RCT designs (Tables 1, 2) (25). In the Heidelberg Classification, PH2 is considered a distinct entity from other ICHs owing to its pronounced impact on functional outcome and death. Details are also provided in the original publication to help the clinician distinguish the types of PH (25).

**Table 1:** Intracranial hemorrhage (ICH) and symptomatic ICH (sICH) rates in intravenous recombinant tissue plasminogen activator (IV tPA) randomized controlled trials.

**RCT**	**Year**	**Treatment**	**Dose**	**Timeframe**	**Safety Outcomes**	**Results**
NINDS part 1	1995	Alteplase	0.9 mg/kg	<3 h 50% within 90 min	CT required at 24 h and 7 to 10 days or when clinically necessary sICH: if not seen on previous CT and any decline in neurologic status	**Asymptomatic ICH** tPA: *N =* 5 (3%); placebo: *N =* 3 (2%) **Symptomatic ICH during the first 36 h** tPA: *N =* 8 (6%); placebo: *N =* 0; *p* < 0.001 *Fatal:* tPA: *N =* 4; placebo: *N =* 0 *Non-fatal:* tPA: *N =* 4; placebo: *N =* 0
NINDS part 2	1995	Alteplase	0.9 mg/kg	<3 h 50% within 90 min	CT required at 24 h and 7 to 10 days or when clinically necessary sICH: if not seen on previous CT and any decline in neurologic status	**Asymptomatic ICH** tPA: *N =* 5 (3%); placebo: *N =* 3 (2%) **Symptomatic ICH during the first 36 h** tPA: *N =* 8 (6%); placebo: *N =* 0; *p* < 0.001 *Fatal:* tPA: *N =* 4; placebo: *N =* 0 *Nonfatal:* tPA: *N =* 4; placebo: *N =* 0
ECASS 1	1995	Alteplase	1.1 mg/kg	0–360 min	CT at 24 h and between day 6 and 8 HI I/II and PH I/II HI I: small petechiae along the margins of the infarct HI II: more confluent petechiae within the infarct area, without space-occupying effect PH I: blood clot <30% of the infarct area with mild space-occupying effect PH II: dense blood clot >30% of the infarct volume with significant space-occupying effect	**Deaths from ICH:** 6.3% in tPA vs. 2.4% in placebo, *p* = 0.02 (ITT analysis) **Overall rates of ICH in ITT analysis:** *N =* 134 in the tPA vs. *N =* 113 in placebo **Overall HI:** (ITT) *N =* 72 in the tPA vs. *N =* 93 in placebo, *p* < 0.001 **Overall PH:** (ITT) *N =* 62 in the tPA vs. *N =* 20 in placebo, *p* < 0.001
ECASS 2	1998	Alteplase	0.9 mg/kg	0–360 min	CT 22–36 h after treatment and at day 7 HI1, HI2, PH1, PH2 Symptomatic and asymptomatic	*Up to day 7* tPA (0–3 h) vs. placebo: HI1: 19% vs. 29% HI2: 12% vs. 16% PH1: 1% vs. 4% **PH2: 7% vs. 1%** tPA (3–6 h) vs. placebo: HI1: 19.9% vs. 23.3% HI2: 16% vs. 11.3% PH1: 4.3% vs. 1.9% **PH2: 8.3% vs. 0.6%** Total rt-PA vs. placebo: HI1: 19.6% vs. 24.3% HI2: 15.2% vs. 12.2% **PH: 11.8% vs. 3.1% (x4 for tPA)** PH1: 3.7% vs. 2.3% **PH2: 8.1% vs. 0.8%** **Frequency of sICH: 2.5-fold excess with tPA**
ATLANTIS A	2000	Alteplase	0.9 mg/kg	0–360 min	CT at 24 h and any CT within 10 days Symptomatic or asymptomatic according to the blinded investigator	Asymptomatic ICH (day 10): tPA 12.7% vs. Placebo 4.3% Symptomatic ICH/fatal ICH (day 10): tPA: 11.3% vs. 0%
ATLANTIS B	1999	Alteplase	0.9 mg/kg	0–300 min	CT at baseline, 18–30 h (sooner if deterioration) Symptomatic or asymptomatic according to the blinded investigator ICH: presence of any blood seen on a brain CT	Asymptomatic ICH: tPA 11.4% vs. placebo 4.7%, *p* = 0.004 Symptomatic ICH: tPA 7% vs. placebo 1.1%, *p* < 0.001 Fatal ICH: tPA 3% vs. placebo 0%, *p* = 0.005
SITS-MOST	2007	Alteplase	0.9 mg/kg	0–180 min	Local or remote PH2 on the 22–36 h posttreatment scan, combined with a neurological deterioration of ≥4 in NIHSS score from baseline, or from the lowest NIHSS score between baseline and 24 h, or leading to death Also, sICH according to the NINDS, ECASS, definitions ICH also assessed on additional scans if needed	At 22–36 h scans - HI1: 5.4% - HI2: 4.0% - PH1: 2.6% - PH2: 2.5% - Remote PH1: 1.7% - Remote PH2: 1.1% sICH rates according to the SITS-MOST definition: - Experienced centers: 1.6% - New centers: 1.7% sICH rates according to the NINDS definition and Cochrane reviews: - Experienced centers: 7.3% - New centers: 7.3%
ECASS 3	2008	Alteplase	0.9 mg/kg	180–270 min	CT or MRI at 22–36 h. Additional CT studies at the discretion of the investigators HI1, HI2, PH1, PH2 sICH: any apparently extravascular blood in the brain or within the cranium associated with clinical deterioration (≥4 increase in NIHSS score or death)	ICH: tPA vs. placebo 27% vs. 17.6%, *p* = 0.001 sICH rates tPA: <3 cases per 100 patients, (2.4), higher compared to placebo: OR = 9.85 (95% CI = 1.26–77.32), *p* = 0.008 *According to ECASS II* definition: OR = 2.43 (1.11–5.35), *p* = 0.02 *According to SITS-MOST:* OR = 7.84 (0.98–63), *p* = 0.02 *According to NINDS:* OR = 2.38 (1.25–4.52), *p* = 0.006 All sICH occurred within 22–36 h
EPITHET	2008	Alteplase	0.9 mg/kg	180–360 min	sICH: ICH with significant clinical deterioration of ≥4 in NIHSS score within 36 h of treatment, AND parenchymal hemorrhage of grade 2 on CT (PH2), SITS-MOST definition	Rates of sICH: tPA 7.7% vs. placebo 0% Median baseline DWI volumes did not differ between patients with without sICH
IST-3	2012	Alteplase	0.9 mg/kg	Within 360 min	sICH: “Clinically important worsening on a valid stroke scale or occurrence of a clinical syndrome suggesting recurrent stroke with ICH on CT/MR performed within 7 days Asymptomatic ICH: ICH within 7 days” Asymptomatic ICH: “presence of any ICH on CT/MR performed within 7 days without any clinical deterioration”	sICH (tPA vs. placebo): Total: OR = 6.94 (4.07–11.8), *p* < 0.0001 Non-fatal: OR = 5.56 (2.72–11.4), *p* < 0.0001 Fatal: OR = 8.12 (3.68–17.9), *p* < 0.0001
ENCHANTED	2016	Alteplase	0.9 vs. 0.6 mg/kg	Within 270 min	Deterioration in neurologic symptoms (≥4 in NIHSS score) and the rates of PH2 on MRI 22 to 36 h after treatment	sICH by SITSMOST definition: low-dose vs. standard dose 1% vs. 2.1%, OR = 0.48 (0.27–0.86), *p* = 0.01 As exploratory analysis, according to the NINDS definition: low-dose vs. standard dose 5.9% vs. 8.0%, OR = 0.73 (0.55–0.95), *p* = 0.02
Wake-Up	2018	Alteplase	0.9 mg/kg	Mismatch DWI/FLAIR	Deterioration in neurologic symptoms (≥4 in NIHSS score) and rates of PH2 on MRI 22 to 36 h after treatment	PH2: tPA vs. placebo 4% vs. 0.4%, OR = 10.46 (1.32–82.77), *p* = 0.03 sICH (SITS-MOST): tPA vs. placebo: 2% vs. 0.4%, OR = 4.95 (0.57–42.87), *p* = 0.15 sICH (ECASS 2): 2.8% vs. 1.2%, OR = 2.40 (0.60–9.53), *p* = 0.21 sICH (ECASS III): 2.4% vs. 0.4%, OR = 6.04 (0.72–50.87), *p* = 0.10 sICH (NINDS): 8% vs. 4.9%, OR = 1.78 (0.84–3.71), *p* = 0.13
ECASS 4/ EXTEND/ EPITHET	2019	Alteplase	0.9 mg/kg	270–540 min	sICH: PH2 within 36 h after intervention with ≥4 increase in NIHSS score from baseline	sICH alteplase: 5% vs. sICH placebo: <1% OR = 9.70 (1.23–76.55), *p* = 0.03
EXTEND	2019	Alteplase	0.9 mg/kg	270–540 min	sICH: PH2 within 36 h after intervention with ≥4 increase in NIHSS score from baseline	sICH tPA vs. placebo: 6.2% vs. 0.9%, aOR = 7.22 (0.97–53.54), *p* = 0.053

*95% CI, 95% confidence interval; ATLANTIS, Alteplase Thrombolysis for Acute Non-interventional Therapy in Ischemic Stroke; CT, computed tomography; DWI, diffusion-weighted imaging imaging; ECASS, European Cooperative Acute Stroke Study; EXTEND, Extending the Time for Thrombolysis in Emergency Neurological Deficits; FLAIR, fluid-attenuated inversion recovery; HI, hemorrhagic infarct; ITT, intention to treat; ICH, intracranial hemorrhage; IV tPA, intravenous recombinant tissue plasminogen activator; IST 3, International Stroke Trial; MRI, magnetic resonance imaging; NIHSS, National Institutes of Health Stroke Scale; NINDS, National Institute of Neurological Disorders and Stroke; OR, odds ratio; PH, parenchymal hematoma; RCT, randomized controlled trial; sICH, symptomatic intracranial hemorrhage; SITS-MOST, Safe Implementation of Thrombolysis in Stroke-Monitoring Study*.

**Table 2:** ICH and sICH rates in endovascular therapy (EVT) randomized controlled trials.

**RCT**	**Year**	**ICH and sICH definitions**	**Results** **EVT+IV tPA vs. IV tPA alone**
MRCLEAN	2015	sICH defined as neurologic deterioration (increase of ≥4 NIHSS score) and evidence of ICH on imaging study Follow-up CT scan at 24 h and 5 days (ECASS)	- Any type of sICH: 7.7% vs. 6.4% - PH2: 6% vs. 5.2% - SAH: 0.9% vs. 0%
ESCAPE	2015	sICH defined as new ICH (ICH, SAH, IVH, or SDH), associated with clinical evidence of neurological worsening, in which the hemorrhage is judged to be the most important cause of the neurological worsening. Clinical worsening guided by the NIHSS score of a minimum of two or more points different from baseline	- sICH: 3.6% vs. 2.7% - Perforation of the middle cerebral artery: 0.6% vs. 0%
EXTEND-IA	2015	- sICH: Any intracranial, including any subarachnoid hemorrhage, associated with clinical symptoms - Symptomatic intracerebral hemorrhage: parenchymal hematoma type 2 (PH2) within 36 h after treatment combined with an increase of ≥4 in NIHSS score from baseline	- sICH: 0% vs. 6% - PH: 11% vs. 9%
REVASCAT	2015	sICH according to the SITS-MOST and ECASS 2 definitions: - PH-2 on follow-up imaging (within 36 h) and neurologic deterioration of ≥4 in NIHSS score. - Any type of intracerebral hemorrhage on posttreatment imaging with an increase of ≥4 in NIHSS score	- sICH according to the SITS-MOST definition: 1.9% vs. 1.9% - sICH according to the ECASS 2 definition: 4.9% vs. 1.9% - Asymptomatic ICH: 16.5% vs. 10.7% - SAH: 4.9% vs. 1.9% - PH2: 2.9% vs. 1.9% - Arterial perforation: 4.9% vs. 0% - Arterial dissection: 3.9% vs. 0%
SWIFTPRIME	2015	sICH assessed at 27 ± 6 h and defined as any PH1, PH2, remote ICH, SAH, IVH associated with ≥4 points worsening in NIHSS score within 24 h	- sICH at 27 hours: 0% vs. 3% - Parenchymal hematoma: 5% vs. 7% - PH1: 4% vs. 3% - PH2: 1% vs. 4% - SAH: 4% vs. 1%
THRACE	2016	sICH at 24 h: visible intracranial bleeding on CT or MRI plus an increase of ≥4 in NIHSS score Asymptomatic ICH on CT or MRI at 24 h	- sICH: 2% vs. 2% - HI1: 10% vs. 12% - HI2: 13% vs. 11% - PH1: 6% vs. 5% - PH2: 7% vs. 4% - SAH: 4% vs. 1% - IVH: 4% vs. 2% - Remote ICH: 2% vs. 3% - Perforation: 1% vs. 0%
DEFUSE 3	2018	sICH defined as an increase of ≥4 in NIHSS score that was associated with brain hemorrhage on imaging within 36 h after stroke onset	*In this trial, the control group did not receive alteplase* - sICH: 7% vs. 4% - PH2: 9% vs. 3%
DAWN	2017	sICH defined as the presence of extravascular blood in the cranium associated with an increase of ≥4 in NIHSS score or death and judged to be the predominant cause of neurologic deterioration within 24 h after randomization (ECASS 3 definition)	*In this trial, the control group did not receive alteplase* - sICH: 6% vs. 3% - Perforation: 0%

*ATLANTIS, Alteplase Thrombolysis for Acute Non-interventional Therapy in Ischemic Stroke; CT, computed tomography; DWI, diffusion-weighted imaging; ECASS, European Cooperative Acute Stroke Study; EVT, endovascular therapy; FLAIR, fluid-attenuated inversion recovery; EXTEND, Extending the Time for Thrombolysis in Emergency Neurological Deficits; HI, hemorrhagic infarct; ITT, intention to treat; ICH, intracranial hemorrhage; IV tPA, intravenous recombinant tissue plasminogen activator; IVH, intraventricular hemorrhage; IST 3, International Stroke Trial; MRI, magnetic resonance imaging; NIHSS, National Institutes of Health Stroke Scale; NINDS, National Institute of Neurological Disorders and Stroke; OR, odds ratio; PH, parenchymal hematoma; RCT, randomized controlled trial; SAH, subarachnoid hemorrhage; SDH, subdural hematoma; sICH, symptomatic intracranial hemorrhage; SITS-MOST, Safe Implementation of Thrombolysis in Stroke-Monitoring Study*.

In summary, several classifications exist for ICH and sICH assessment, often originating from a dedicated RCT. Depending on the aforementioned definitions, these classifications are more or less inclusive and may capture varying degrees of bleeding events after AIS treated with IV tPA. For instance, the NINDS and International Stroke Trial 3 (IST-3) definitions are considered inclusive because they assess ICH within 7 days, with all types of ICH considered. In contrast, the SITS-MOST definition is more restrictive (PH2), with specific clinical deterioration definitions and shorter timeframe (22–36 h). These discrepancies regarding ICH definitions may explain, at least in part, the variations in ICH rates seen among trials, which is discussed below.

### Results From IV tPA RCTs

#### IV tPA Therapy Increases ICH Rates and Severity After an AIS

With now 25 years since the original publication of the NINDS trial and the consequent publication of results of several large RCTs evaluating different tPA molecules in the setting of AIS, we have a large amount of data regarding ICH rates after IV tPA therapy.

In the NINDS trial, 3% of the tPA group had asymptomatic ICH as compared with 2% in the placebo group (6). However, 6% of patients had sICH during the first 36 h in the tPA group, 50% of whom died, as compared with none in the placebo group (*p* < 0.001).

In the ECASS 3 trial, ICH rates (i.e., with the radiological definitions described above) reached 27% in the tPA group vs. 17.6% in the placebo group (*p* = 0.001) (3). sICH occurred in 2.4% vs. 0.2%, respectively, with tPA treatment leading to an increase in sICH rate as compared with the placebo group (OR = 9.85, 95% CI = 1.26–77.32, *p* = 0.008) (3). Of note, the ECASS 3 investigators also assessed sICH according to different validated definitions used in prior trials. This additional analysis is instructive because it highlights the previously discussed issue of ICH and sICH definitions. Therefore, in ECASS 3, sICH occurred in 5.3% and 2.2% of the tPA and placebo groups, respectively, according to the ECASS 2 definition (OR = 2.43, 95% CI = 1.11–5.35, *p* = 0.02), 1.9 and 0.2% according to the SITS-MOST definition (OR = 7.84, 0.98–63, *p* = 0.02), and 7.9 and 3.5% according to the NINDS definition (OR = 2.38, 1.25–4.52, *p* = 0.006) (3). All sICH cases in the ECASS 3 trial occurred within 22 to 36 h.

In the SITS-MOST study, PH1 and PH2 on the 22 to 36 h posttreatment CT scan occurred in 2.6 and 2.5% of patients, respectively (31). According to the SITS-MOST definition, sICH occurred in 1.6 and 1.7% of patients in experienced and new SITS-MOST centers, respectively (0.28% fatal within 24 h) and reached 2.2% within 7 days (31). According to the NINDS definition, 7.3% had sICH, which underlines the higher ICH rates with the NINDS definition (31).

In the IST-3 trial, tPA treatment yielded a 7-fold significantly increased number of sICH cases and an 8-fold increase in fatal sICH cases (33).

In 2004, a first pooled analysis of the Alteplase Thrombolysis for Acute Non-interventional Therapy in Ischemic Stroke (ATLANTIS), ECASS and NINDS trials showed a substantial increase in PH2 rates in the tPA group as compared with the placebo group (5.9% vs. 1.1%), 60% being fatal within 3 months (34). These hematomas were associated with tPA treatment and age but not baseline NIHSS score or time from stroke onset to IV tPA infusion (34). Of note, and as previously described, substantial differences existed between the trials in the definition of ICH.

In 2012, Wardlaw et al. performed an additional updated systematic review and meta-analysis of all evidence from RCTs for IV tPA in AIS (35). This study yielded 12 RCTs including more than 7,000 patients with IV tPA given within 6 h from stroke onset. Data for fatal ICHs were available from eight trials and ICHs occurred in 3.6% of the tPA group vs. 0.6% of the placebo group, with an absolute increase of 29 deaths/1,000 patients (35). Moreover, tPA use did not increase the number of deaths within 7 days from causes other than ICH, which underlines a strong association between early deaths in the tPA group and fatal ICH (35). All 12 trials assessed sICH, which occurred in 7.7% of the tPA group vs. 1.8% of the placebo group (35). This resulted in a crude estimate of the absolute excess of sICH with tPA of 58/1,000 patients treated (35). Finally, in 2014, a meta-analysis of individual patient data from nine trials (5) evaluated sICH with the ECASS 3 and SITS-MOST definitions: tPA significantly increased the likelihood of sICH. Among the 3,391 patients receiving tPA, 231 (6.8%) had a PH2 within 7 days vs. 44 (1.3%) in the control group (OR = 5.5, 95% CI = 4.01–7.70) (5). Similar results were obtained for SITS-MOST–criteria PH2 within 36 h (OR = 6.67, 95% CI = 4.11–10.84) (5). These PH2 cases were fatal within 7 days for 2.7% patients in the tPA group vs. 0.4% in the control group. Importantly, this early excess mortality caused by ICH in the tPA group did not result in increased overall mortality at 3 months and did not limit tPA effectiveness on 3 month functional outcomes (5).

In 2016, the Enhanced Control of Hypertension and Thrombolysis Stroke Study (ENCHANTED) evaluated the non-inferiority of a lower dose of IV tPA (0.6 mg/kg) compared to the standard dose (0.9 mg/kg) (36). Although ENCHANTED did not show the non-inferiority of low-dose IV tPA to standard dose with respect to death and functional outcomes at 3 months, the low-dose group had significantly fewer sICHs. With the SITS-MOST definition, the frequency of sICH was lower in the low-dose than standard-dose group (1% vs. 2.1%; OR = 0.48, 95% CI = 0.27–0.86) (36). sICH rates with the NINDS definition were also lower in the low-dose group. In addition, fatal ICH rates were lower in the low-dose than standard-dose group (1.3% vs. 2.5%) (36). These results are particularly relevant and support the impact of IV tPA dose as a risk factor for sICH. Nevertheless, given the non-significant results, current North American and European guidelines recommend a standard dose for IV tPA treatment, considered as the best benefit–risk ratio (1, 2).

The Efficacy and Safety of MRI-based Thrombolysis in wake-up Stroke trial evaluated magnetic resonance imaging (MRI)–guided thrombolysis for stroke with unknown time onset (37). sICH occurred in 2% of patients in the IV tPA group and 0.4% in the placebo group (adjusted OR = 4.95, 95% CI = 0.57–42.87), according to the SITS-MOST definition (37). These proportions remained similar according to the ECASS 2, ECASS 3 and NINDS definitions (37). Recently, the EXTEND trial assessed the effectiveness of IV tPA initiated 4.5 to 9.0 h after stroke onset in patients with a favorable perfusion-imaging profile detected by automated perfusion imaging (38). sICH occurred in 6.2% of patients in the IV tPA group and 0.9% in the placebo group (adjusted relative risk=7.22, 95% CI = 0.97–53.54) according to the SITS-MOST definition (38). A recent systematic review and meta-analysis of individual data from the EXTEND, ECASS-4 and EPITHET trials, all evaluating the effectiveness of IV tPA beyond 4.5 h based on perfusion mismatch, found a frequency of 5% sICH in the IV tPA group vs. <1% in the placebo group (OR = 9.7, 95% CI = 1.23–76.55) (32), with the SITS-MOST definition used for this study. Hence, the frequency of sICH beyond 4.5 h in patients receiving IV tPA seems similar to that with the 0 to 4.5 h window, with a 3.4% sICH frequency according to the SITS-MOST definition in the meta-analysis of individual data published by Whiteley et al. (39). Fatal ICH occurred in 2.3% of patients receiving IV tPA beyond 4.5 h (32) and 2.6% receiving IV tPA from 0 to 4.5 h (39). Despite indirect comparisons between the 0 to 4.5 and 4.5 to 9.0 h time windows, these data seem to show the overall safety of IV tPA beyond 4.5 h in patients selected with a favorable perfusion-imaging profile regarding sICH rates. Similarly, the safety profile regarding sICH proportion in “wake-up stroke” patients receiving IV tPA is also reassuring and should favor IV tPA implementation, if indicated.

In summary, the results from RCTs and meta-analyses including individual pooled patient data point in the same direction and demonstrate an increased risk of PH2 and sICH after tPA treatment by 6-fold [depending on the definition used (39)], responsible for excess early mortality caused by ICH. Despite indirect comparisons, these results seem similar for the 4.5 to 9.0 h time window (32, 38). Nevertheless, and despite these concerning findings, the beneficial effect of tPA on 3 month functional outcome remained significant among these studies. From a practical viewpoint, it seems crucial to evaluate the ICH risk in different subgroups of patients and to assess the impact of different suspected or known risk factors for ICH after IVT. This analysis, discussed below, could pave the way toward individualized management.

### Predictive Factors of ICH Occurrence

A recent meta-analysis of individual patient data found a proportional increase in ICH risk in patients receiving tPA regardless of pre-specified subgroups such as age (≤80, >80 years old), treatment delay (≤3, 3–4.5, >4.5 h) and baseline stroke severity (NIHSS score 0–4, 5–10, 11–15, 16–21, ≥22), with non-significant interaction (39). Several previous publications attempted to outline risks factors for PHs or sICH. However, these works are difficult to interpret given the different ICH definitions used (16, 17, 40). In the ECASS 1 trial, risk factors for PHs increased with age and IV tPA treatment (17, 40). In the NINDS trial, factors associated with sICH were baseline neurological severity, the presence of early ischemic changes on pretreatment brain CT scan and IV tPA treatment (6). In the ECASS 2 trial, significant risk factors for PHs were IV tPA treatment, extent of parenchymal hypodensity on baseline CT scan, history of congestive heart failure, increasing age and a high baseline systolic blood pressure (40). Of note, two interactions were detected: IV tPA with age and aspirin, meaning that PHs after IV tPA treatment was more frequent in older patients and in patients receiving aspirin at the time of stroke (40). The evidence that aspirin could increase the sICH risk is of interest and was confirmed by the Early Administration of Aspirin In patients treated with Alteplase for Acute Ischemic Stroke (ARTIS) trial (41). In this trial, sICH occurred more often in the aspirin than control group (4.3 and 1.6%) and was responsible for the poor outcome in the aspirin vs. control group (41). This finding was also confirmed by a *post hoc* analysis showing that aspirin increased the risk of early neurologic deterioration due to sICH by a factor of 3.7 in patients receiving tPA (42). In the SITS-IST registry (SITS-ISTR), baseline anti-thrombotic treatment was the most pronounced risk factor, with the association of aspirin and clopidogrel increasing by 3-fold and aspirin alone by 2-fold the odds of sICH occurrence (43). Baseline neurological severity with NIHSS score ≥13 was independently associated with sICH (OR = 2.2, 95% CI = 1.7–3.0), as was NIHSS score 7 to 12 (OR = 1.6, 95% CI = 1.1–2.1). Other factors were baseline glucose level (≥180 mg/dL), age ≥72 years, systolic blood pressure ≥146 mmHg, weight ≥95 kg, onset-to-treatment time ≥180 min and history of hypertension (43). Baseline neurological severity was also associated with absolute excess risk of PH2, fatal ICH, and sICH according to the SITS-MOST definition in a secondary analysis of individual data from a meta-analysis (39). In this analysis, with baseline NIHSS score 0 to 4, the absolute excess risk of ICH in the tPA group over the control group was 1.5% (95% CI = 0.8–2.6) as compared with 3.7% (95% CI = 2.1–6.3) with baseline NIHSS score ≥22 (39).

As illustrated above, several risk factors for ICH after IV tPA seem to emerge across studies and deserve specific attention.

#### Age

Evidence regarding the impact of age as a risk factor for ICH are conflicting. Several studies have showed such associations, as in the ECASS 1 trial, in which each 10 year increase in age predicted PHs (16). In the pooled analysis of the ATLANTIS, ECASS, and NINDS trials, secondary analysis showed age is strongly associated with the occurrence of PHs (*p* = 0.0002) (34). In a meta-analysis of 55 studies, Whiteley et al. found an association between older age and significantly increased risk of sICH after IV tPA; however, this study did not include the IST-3 trial data (44). Of note, other studies seemed to show the opposite, especially at the 80 year-old threshold (45, 46). Mishra et al. found that the rate of sICH according to the SITS-MOST definition was 2.5% vs. 1.9% with age ≥80 vs. <80 years and thus not significantly higher (OR = 1.3, 95% CI = 0.96–1.8, *p* = 0.07) (47). According to the NINDS definition (any increase in NIHSS score from baseline and any parenchymal ICH), the corresponding rate of sICH was significantly higher (11% vs. 8.3%), thus highlighting the importance of the sICH definition, as mentioned previously (47). Likewise, Ford et al. found that the unadjusted sICH rate did not differ significantly between patients >80 and ≤80 years old with the SITS-MOST definition (1.8% vs. 1.7%) but was again significantly increased according to the NINDS definition (48). Multivariate analysis adjusting for several baseline prognostically important factors found no difference in sICH rates between patients >80 and ≤80 years old (OR = 0.90, 95% CI = 0.73–1.09 according to the SITS-MOST definition and OR = 0.96, 95% CI = 0.87–1.06 according to the NINDS definition) (48). In the Multicenter rt-PA Acute Stroke Survey, age (per 10 years) was associated with sICH (OR = 1.32, 95% CI = 1.03–1.69) but did not remain significant on adjustment for clinical, biological, and radiological cofounders (49). In a systematic review, Engelter et al. found a similar likelihood of sICH in patients ≥80 and <80 years old (50). Unfortunately, limited data from RCTs are available because age ≥80 years was usually considered an exclusion criterion. In the IST-3 trial, almost 50% of the randomized patients were >80 years old, including 7% who were >90 years old, thus providing important data regarding IV tPA safety in this population (33). An updated meta-analysis found no heterogeneity regarding fatal ICH (*p* = 0.4) and sICH (*p* = 0.1) in the IST-3 trial and other trials included in the study (35). Patients >80 years old seemed to benefit from IV tPA, especially up to 3 h after stroke onset (35). Altogether, these findings highlight the importance of sICH definition (e.g., SITS-MOST vs. NINDS definition). In addition, the coexistence of numerous comorbidities in older people could explain the increased risk of sICH in some studies. Thus, age could become an indirect marker of other relevant factors associated with sICH, notably, the coexistence of a microangiopathy, such as cerebral amyloid angiopathy, highly prevalent in this population. This issue is discussed below with radiological predictive factors of ICH.

#### Stroke Severity

Baseline neurological severity was one of the three risk factors independently associated with sICH in the NINDS trial (five NIHSS categories, OR = 1.8, 95% CI = 1.2–2.9) (29). In the Multicenter t-PA Acute Stroke Survey, baseline NIHSS (per one-category increase) was also a risk factor for all tPA-related ICH and remained an independent predictor in each multivariate statistical model (49). These results were also confirmed in an individual-patient data meta-analysis, which found an absolute excess risk of PH2, fatal ICH, and sICH according to the SITS-MOST definition with increasing baseline stroke severity (39). Because NIHSS scores are closely related to infarct volume, these findings could also result from the association between sICH risk and baseline extensive infarct lesions (51). In addition, risk for sICH is non-linear with NIHSS score ≥20 and may actually decrease with NIHSS score >25 (52). This finding is likely explained by “ceiling effects” of the NIHSS, particularly for basilar artery occlusion, which may be associated with severe disturbance of consciousness and high NIHSS score while causing ischemia in a tissue volume less than that of a complete middle cerebral artery territory (52). Recently, the risk of sICH after IV tPA in posterior circulation strokes was found to be half of that of anterior circulation strokes, albeit with higher risk of death, which illustrates the limitations of the NIHSS score for stroke severity assessment (53).

#### Blood Pressure

Hypertension has major consequences both during the early and late phases of AIS (54). The IST found no association between baseline systolic blood pressure and sICH (55). However, in the same year, mean blood pressure (per 25 mmHg) was found independently associated with all tPA-related ICH in the multicenter rt-PA Acute Stroke Survey (49). Prebolus systolic blood pressure >185 mmHg and diastolic blood pressure >110 mmHg were independently associated with increased odds of sICH (OR = 2.59, 95% CI = 1.07–6.25) (56). These results were also confirmed within the first 24 h after IV tPA in the SITS-ISTR (57). Indeed, high systolic blood pressure 2 to 24 h after IV tPA as a categorical variable was linearly associated with sICH occurrence (57). The EPITHET investigators also found a strong association between post-tPA blood pressure and PH rates (58). The 24 h weighted average systolic blood pressure predicted PH, and for every 10 mmHg increase in blood pressure post-treatment, the odds of PHs increased by 59% (58). In the IST-3, the odds of sICH also increased by 10% with each 10 mmHg increase in baseline systolic blood pressure (59). In this study, systolic blood pressure variability (i.e., standard deviation) was associated with 3 month death but not sICH. This concept of blood pressure variability after IV tPA is critical and has been reported to have an independent association with infarct growth, early clinical course, and 3 month functional outcome (60). The SITS investigators confirmed these findings and found a strong association between blood pressure variability defined by successive variation within the first 24 h and the occurrence of sICH according to either the SITS-MOST or ECASS definitions (61).

The ENCHANTED trial also assessed intensive blood pressure–lowering (target systolic blood pressure 130–140 mmHg within 60 min of randomization) as compared with guideline-recommended blood pressure lowering (target systolic blood pressure <180 mmHg) in patients receiving IV tPA for AIS (62). In this study, the frequency of any ICH (within 7 days after randomization) was lower in the intensive blood pressure–lowering group than the guideline-recommended blood pressure group (14.8% vs. 18.7%, *p* = 0.01). However, sICH defined by the SITSMOST, NINDS, ECASS 2, ECASS 3, and IST-3 definitions did not significantly differ among the two groups, despite a trend for lower frequency in the intensive blood-pressure management group (62). Similarly, rates of PH2 and fatal ICH within 7 days were lower but not significantly in the intensive blood pressure–lowering group as compared with the guideline-recommended group (62). Overall, these findings suggest that the blood-pressure control strategy in the early management of AIS may affect the occurrence of hemorrhagic complications. However, because of statistically non-significant results, the optimal blood pressure target for preventing ICH after IV tPA is currently not defined, and further studies are needed to answer this question.

#### Baseline Glycemia and Diabetes Mellitus

Hyperglycemia and diabetes mellitus are known risk factors for ICH and sICH. Since the early 2000s, the Multicenter rt-PA Stroke Survey Group showed a nearly 4-fold increase in risk of sICH after IV tPA in diabetic patients (49). As well, glucose level (per 50 mg/dL increase) was strongly associated with all ICHs in this study (OR = 1.36, 95% CI = 1.11–1.67) (49). SITS investigators confirmed these findings and found a 2-fold increase in sICH risk for patients with baseline glucose level ≥180 mg/dL (43). In a systematic review and meta-analysis of 54 studies, diabetes was associated with increased odds of sICH in unadjusted analysis but not multivariate analysis (63). Increased admission glucose level was also associated with a 9% increase in sICH on multivariate analysis (63). Of note, pathophysiological processes triggered by hyperglycemia and responsible for ICH seem mediated by microvascular thromboinflammation exacerbation, with an increased activation of platelets and neutrophils (64).

#### Ethnicity

Ethnicity could also be a risk factor for sICH after reperfusion therapy as is clearly the case for acute ST segment elevation myocardial infarction (65). Numerous publications have assessed the effectiveness and safety of IV tPA in the Asian population, which presents unique aspects of AIS, such as higher frequency of intracranial atheroma and intracranial dissection (66). Of note, the official dose of IV tPA in Japan and several countries in Eastern and Southern Asia is 0.6 mg/kg, which complicates the comparison of sICH with European or North American rates (66). However, Toyoda et al. recently compared sICH rates after IV tPA among different registries. The sICH frequency was 1.7% in the European SITS-MOST registry, 1.9% in the SITS-NEW registry (including Korea, China, India and Singapore), 3.5% in the J-MARS registry (Japan), and 1.3% in the SAMURAI r-tPA registry (Japan) (66). Overall, these results are reassuring regarding the safety of IV tPA in the Asian population. Recently, the RADIANT study assessed the impact of racial disparities in ICH occurrence after IV tPA (67). Two ethnic groups (non-Hispanics and Hispanics) and four racial groups [Asian, Black, White, and other (Native Americans, Pacific Islanders)] were identified, and sICH rates did not differ among the racial or ethnic groups (67). Using the Get With the Guidelines Stroke Program, Mehta et al. assessed in 54,334 patients the impact of race/ethnic-related differences on safety outcomes after IV tPA (68). This study found a higher rate of hemorrhagic complications after IV tPA in Black (OR = 1.14, 95% CI = 1.04–1.28) and Asian patients (OR = 1.36, 95% CI = 1.14–1.61). This increased risk was due to sICH in Asian patients but risk of other bleeding in Black patients (68). Several reasons may contribute to this result: differences in health care access regarding vascular risk factor prevention and genetic variants among these populations.

#### Neuroradiological Findings

This section raises the question of the imaging modality performed before IV tPA therapy in the setting of AIS. As the validated imaging modality, brain CT, is fast and provides the required information needed for the medical management in the acute phase but ultimately provides few imaging ICH predictive factors, only the early CT changes >33% of the middle cerebral artery territory before IV tPA were found a predictor of sICH and increased the odds of sICH by 6.7 (49). Among the different MRI patterns possibly associated with increased odds of sICH, several studies highlighted the prognostic value of baseline apparent diffusion coefficient and the diffusion-weighted lesion volume (69–71). Still, the presence of cerebral microbleeds (CMBs) seems to be the main radiological finding strongly associated with risk of sICH. A recent meta-analysis showed that the presence of CMBs and high CMB burden on baseline MRI were independently associated with sICH (72). In a recent individual-patient data meta-analysis, patients with CMB showed increased risk of PHs and remote PHs but not sICH (73). CMB burden was associated with sICH and PH, and >10 CMBs independently predicted poor functional outcome (73).

#### Blood Biomarkers

Several studies assessed the association between baseline blood cell count and sICH occurrence in AIS patients receiving IV tPA. Baseline neutrophil count and neutrophil-to-lymphocyte ratio were independently associated with increased risk of sICH (74, 75). Other studies found baseline low platelet count associated with increased sICH risk (76, 77).

Finally, two studies assessed the impact of early fibrinogen degradation coagulopathy after IV tPA therapy. Both found an independent increased risk of ICH associated with an early decrease in fibrinogen level. These last results support IV tPA–induced fibrinogen degradation, with its biological consequences on hemostasis and platelet aggregation, as one of the main factors in post-AIS ICH severity (15, 78).

## ICH After Endovascular Therapy

Since 2015, the association of EVT and IV tPA is the gold-standard treatment of AIS consecutive to LVO of the anterior circulation (4). This approach has revolutionized AIS management, leading to >80% successful reperfusion after treatment and improved 3 month functional outcomes (4). The assessment of hemorrhagic complications in the context of EVT is recent and must acknowledge certain features that were irrelevant for IV tPA alone, such as the high rates of reperfusion, but also possible complications due to EVT devices and navigation in the intracranial arteries.

### Results From RCT and Observational Studies

As illustrated in Table 2, the association of EVT and IV tPA did not increase the rate of PH2 or sICH vs. IV tPA alone. However, comparing results of trials is challenging, given the different definitions of sICH used. In 2016, a meta-analysis of individual patient data from five RCTs evaluated the proportion of patients with sICH as defined by each trial and PH2 rates within 5 days (4). Overall, sICH rates did not differ between the intervention group (EVT and IV tPA, 4.4%) and control group (IV tPA alone, 4.3%) (4). These results were also confirmed for the rate of PH2 (5.1% in the intervention group vs. 5.3% in the control group) (4). Another recent meta-analysis of patient-level data assessed the safety features of EVT from seven RCTs and confirmed no difference between sICH and PH2 rates among EVT and control groups (79). PH2 rates showed no treatment effect modification by baseline imaging features. However, EVT was associated with increased odds of sICH for patients with baseline large AIS defined as early ischemic change in >33% of the middle cerebral artery territory (adjusted OR = 4.17, 95% CI = 1.30–13.44) as compared with controls (79). Onset-to-reperfusion time seems a strong predictor of ICH in EVT-treated patients (80). Indeed, in this study, which pooled data from previous EVT trials of first-generation EVT devices, the adjusted OR for each 30 min increase in time was 1.21 (95% CI = 1.10–1.33) for ICH (according to the ECASS 2 definition) (80). These results were also confirmed in the recent meta-analysis of individual patient data from five RCTs, in which the onset-to-reperfusion time was significantly associated with PH2 occurrence (OR per 1 h delay: 1.43 (1.09–1.88) (81).

Observational studies, reflecting real-life practice, have confirmed these data. In a large observational study from the Endovascular Treatment for Ischemic Stroke (ETIS) registry, independent predictors of PHs after EVT included age (per 1 year increase), current smoking, admission ASPECTS (per 1-point decrease), general anesthesia, angiographic poor collaterals, and embolization in a new territory (9). The Multicenter Randomized Clinical Trial of Endovascular Treatment for Acute Ischemic Stroke in the Netherlands (MRCLEAN) investigators performed a similar study and found only increased systolic blood pressure and atrial fibrillation associated with PH occurrence (82). Of note, increased systolic blood pressure and antiplatelet use were also associated with increased odds of sICH (82). Thus, consistent with IV tPA, systolic blood pressure appears to be a predictor of sICH after EVT as well and needs to be taken into account, being an eligible modifiable risk factor (discussed below).

Recently, Nogueira et al., in a multicenter retrospective analysis of 1,122 patients receiving EVT for anterior circulation strokes, assessed the predictors and clinical relevance of hemorrhagic transformation (83). PH occurred in 8.5% and was associated with atrial fibrillation (OR = 1.61, 95% CI = 1.01–2.55), a finding already described in patients receiving IV tPA (83). The use of the MERCI thrombectomy device (a first-generation EVT device) was associated with increased rates of HIs (OR = 1.47, 95% CI = 1.02–2.12), potentially implicating the type of device used during EVT in the occurrence of sICH (83). In the Contact Aspiration vs. Stent Retriever for Successful Revascularization trial, PH2 frequency was greater in the stent retriever group than aspiration group (7.6% vs. 3.7%), but sICH rates were not statistically different (6.5 and 5.3%) (84). In addition, subarachnoid hemorrhages, a known complication of EVT, occurred in 7.1 and 6.9% of the stent retriever and aspiration groups (84). These results were confirmed by the Aspiration thrombectomy vs. stent retriever thrombectomy as first-line approach for LVO trial, with similar sICH rates according to the SITS-MOST definition (3%) (85). Altogether, these results suggest that sICH rates are not increased by EVT and that last-generation devices (i.e., stent retriever and aspiration) have similar sICH and subarachnoid hemorrhage rates.

Although most trials assessed the effectiveness of EVT for LVO (intracranial internal carotid artery, M1 segment of the middle cerebral artery), EVT for M2 occlusion is increasingly performed and raises the question of safety outcomes. In a multicenter retrospective cohort with isolated M2 occlusions, sICH rates did not differ between the EVT and medical groups (86). As compared with M1 occlusions treated with EVT, rates of sICH were lower in the M2 group, notably regarding PH2 rates (87). Overall, these data are reassuring and illustrate the safety of EVT for M2 occlusions in terms of sICH rates.

Finally, because increased time from stroke onset to reperfusion is associated with ICH (80), the “drip and ship” paradigm (i.e., patient transfer from a primary to a comprehensive stroke center for EVT) could be associated with increased risk of sICH as compared with the “mothership” paradigm (primary management in the comprehensive stroke center with EVT). However, despite longer onset-to-recanalization time in the former vs. latter group (292 vs. 240 min), a recent study found similar sICH rates among the two groups (2% vs. 3%, *p* = 0.6) (88). sICH rates were also recently evaluated between directly admitted and transferred patients with data from a national database (8,533 patients) (89): hemorrhage rates did not differ between the two groups, despite longer times from stroke onset to reperfusion (89).

### The Issue of Blood Pressure in EVT-Treated Patients

Numerous studies have recently highlighted the impact of systolic blood pressure in the early phase of AIS treated by EVT (90–93). The MRCLEAN investigators have found higher systolic BP before EVT associated with increased probability of sICH (94). The odds of sICH increased 21% for every 10 mmHg increase in blood pressure (94). In the ETIS registry, baseline systolic blood pressure was also strongly associated with 3 month mortality and functional outcomes (93). sICH rates were not significantly related to baseline systolic or diastolic blood pressure (93). Systolic, pulse pressure and mean arterial BP variability during EVT seem to be critically associated with functional outcome (91, 92, 95–98), but data are scarce regarding the association of blood pressure variability during EVT and sICH risk. After EVT, and notably after complete reperfusion, blood pressure variability seems associated with increased rates of sICH (99, 100). Recently, Matusevicius et al. assessed the association between blood pressure after EVT and sICH based on the recanalization status in a large international registry (101). Increased systolic blood pressure was associated with sICH in patients with unsuccessful recanalization, and mean 24 h systolic blood pressure >160 mmHg was associated with sICH, regardless of the recanalization status (101). These studies are limited by the heterogeneity in definition of sICH, as discussed above, and further research is needed to fully understand the mechanisms underlying these associations.

## Future Directions: How to Decrease Hemorrhagic Complications After Reperfusion Therapy

### Clinical Studies to Come

In the hemodynamic field, the upcoming results of the BP TARGET trial (NCT03160677) are much awaited (102). This RCT with blinded endpoint evaluated the impact on ICH rates of an intensive blood pressure–lowering strategy (systolic blood pressure <130 mmHg) for 24 h after successful reperfusion in AIS patients receiving EVT (102). With ICH rates as the primary outcome, the BP TARGET trial should provide new insights into the impact of blood-pressure management on ICH occurrence. Other RCTs evaluating blood pressure–lowering strategies after reperfusion therapies are currently in progress or are about to begin and include the BEST II trial (NCT04116112), OPTIMAL-BP (NCT04205305), and ENCHANTED 2 (NCT04140110).

In the antithrombotic field, current strategies aim at optimizing cerebral reperfusion pharmacologically as an adjunct to current recommended therapies with IVT and EVT. In this context, ACT017, an antibody fragment targeting platelet GPVI receptor, seems to be a new antiplatelet agent that safely improves reperfusion (103). After showing its safety profile in preclinical and phase I studies, the ACTIMIS trial (NCT03803007) is currently evaluating this first-in-class antiplatelet agent following tPA within 4.5 h from stroke onset. The ACTIMIS trial will be an opportunity to assess the benefit/risk ratio of this new antiplatelet molecule.

Several trials are evaluating the effectiveness and safety of EVT in large ischemic core at presentation. As previously stated, large ischemic core (>33% of the middle cerebral artery territory, low ASPECTS) may expose the patient to increased risk of sICH after reperfusion therapy. The Large Stroke Therapy Evaluation study from the INEXTREMIS Trial (NCT03811769) is evaluating EVT in large ischemic core (ASPECT <5 or 3 to 5 for patients >80 years old). Other studies, such as the Efficacy and Safety of Thrombectomy in Stroke With Extended Lesion and Extended Time Window (TENSION; NCT03094715) and Thrombectomy for Emergent Salvage of Large Anterior Circulation Ischemic Stroke (TESLA; NCT03805308) trials, are also evaluating these outcomes in large ischemic core at presentation. The results of these trials are awaited because they will provide valuable insights regarding the safety of reperfusion treatments in these settings and the impact of reperfusion in patients presenting low ASPECTS.

Finally, because IV tPA is strongly associated with hemorrhagic complications such as sICH (despite its effectiveness) and because EVT results in high (>90%) recanalization rates, several trials are evaluating the non-inferiority of EVT alone vs. the association of IV tPA and EVT in patients directly transferred to a comprehensive stroke center. These trials are based on the hypothesis that for directly admitted patients, IV tPA may increase intracranial bleeding complications without improving reperfusion rates. Two trials [MRCLEAN NO-IV and Bridging Thrombolysis vs. Direct Mechanical Thrombectomy in Acute Ischemic Stroke (SWIFTDIRECT)] are underway and should answer these issues. Results of the Direct Intra-arterial Thrombectomy in Order to Revascularize AIS patients With LVO Efficiently in Chinese Tertiary Hospitals (DIRECT MT) trial were published in 2020 and revealed that EVT alone was non-inferior to EVT after IV tPA (77). The trial found lower, although not significant, sICH rates according to the Heidelberg definition in the direct EVT group than the IV tPA and EVT group (4.3% vs. 6.1%) (77). The results of the other trials are expected, in order to compare the sICH rates between EVT alone and the association of IV tPA and EVT.

## Concluding Remarks

Overall, this review illustrates how ICH is the most feared complication in the early management of AIS. sICH is strongly associated with 3 month unfavorable outcomes and mortality. Importantly, because of the many different definitions of sICH, comparison between studies is difficult. In addition, many sICH risk factors have been identified and include blood biomarkers and clinical and neuroradiological factors, some of which are used in predictive scores (104, 105). However, these scores have so far limited impact on clinical practice, notably because of the lack of effective treatments of sICH. Moreover, despite this known inherent risk of sICH after IV tPA, the benefit–risk ratio favors its use, and no score should restrict its use if indicated. Current ongoing trials aim at extending the indications of EVT (timeframes, occlusion sites, infarct core volume, etc.), improving recanalization rates, while reducing the risk of sICH. From this perspective, many RCTs are of interest because they address the issue of important risk factors of sICH such as blood pressure or antithrombotic treatments. Still, an effort must be made in the design of future RCTs regarding sICH definitions, to improve the implementation of the results in clinical practice and allow for their comparison.

## Author Contributions

BM, JPD, and MM contributed to the review, concept and design, the analysis and interpretation of data from the literature search, drafted the manuscript, made critical revision of the manuscript for important intellectual content. MM supervised this work. All authors read and approved the final manuscript.

## Conflict of Interest

The authors declare that the research was conducted in the absence of any commercial or financial relationships that could be construed as a potential conflict of interest.

## Abbreviations

AIS: acute ischemic stroke
ASPECTS: Alberta Stroke Program Early CT Score
EVT: endovascular therapy
CT: computed tomography
BP: blood pressure
SBP: systolic blood pressure
ICH: intracranial hemorrhage
sICH: symptomatic intracranial hemorrhage
HI: hemorrhagic infarctions
PH: parenchymal hematoma
NIHSS: National Institutes of Health Stroke Scale
mTICI: modified Thrombolysis In Cerebral Ischemia
RCT: randomized controlled trial
IV tPA: intravenous recombinant tissue plasminogen activator.
